# Supplementary material for: Efficient mRNA delivery to resting T cells to reverse HIV latency
Source: Nat Commun. 2025 May 29;16:4979. doi: 10.1038/s41467-025-60001-2 (PMC12122926; doi:10.1038/s41467-025-60001-2)
Supplement: Supplementary file 1 — Supplementary Information [file 41467_2025_60001_MOESM1_ESM.docx]

# SUPPLEMENTARY INFORMATION

**Table S1. Characterisation of LRA-LNP X**. Z-average (hydrodynamic diameter), mean size determined by Number distribution and LNP size uniformity (polydispersity index, PDI) as determined by dynamic light scattering for the LNP X formulation encapsulating mRNA for encoding mCherry, HIV Tat, L+O CRISPRa or scr CRISPRa. mRNA encapsulation efficiency for each formulation was determined using a modified RiboGreen assay. Mean ± SEM for n=3 LNP batches for each formulation.

|  | **mCherry** | **Tat** | **L+O CRISPRa** | **scr CRISPRa** |
| --- | --- | --- | --- | --- |
| **Z-average (d.nm)** | 111.0±4 | 110.9±7 | 116.1±7 | 109.2±12 |
| **Size by Number distribution (d.nm)** | 81.3±6 | 77.2±5 | 83.6±6 | 77.0±12 |
| **PDI** | 0.09±0.03 | 0.10±0.02 | 0.09±0.03 | 0.12±0.01 |
| **Encapsulation efficiency (%)** | 97.4±1 | 96.8±1 | 96.4±1 | 96.1±1 |

# Table S2. HIV LTR-targeting gRNA sequences and positions with LTR. Guide RNA (gRNA) sequences are shown in 5’ to 3’ orientation, including a 20-nt spacer sequence (underlined) and a subsequent 141-nt gRNA scaffold including hairpin structures to attract MS2-p65-HSF1. The position of the protospacer-adjacent motif (PAM) sequence (NGG, where N is any nucleotide) corresponding to each gRNA is shown relative to the HIV transcription start site (TSS). scr, scrambled gRNA.

|  | **Sequence** | **Position to TSS** | **Orientation** |
| --- | --- | --- | --- |
| **gRNA B** | CCA CGU GAU GAA AUG CUA GGG UUU UAG AGC UAG GCC AAC AUG AGG AUC ACC CAU GUC UGC AGG GCC UAG CAA GUU AAA AUA AGG CUA GUC CGU UAU CAA CUU GGC CAA CAU GAG GAU CAC CCA UGU CUG CAG GGC CAA GUG GCA CCG AGU CGG UGC UUU UU | -180 | Reverse |
| **gRNA C** | UGC UAC AAG GGA CUU UCC GCG UUU UAG AGC UAG GCC AAC AUG AGG AUC ACC CAU GUC UGC AGG GCC UAG CAA GUU AAA AUA AGG CUA GUC CGU UAU CAA CUU GGC CAA CAU GAG GAU CAC CCA UGU CUG CAG GGC CAA GUG GCA CCG AGU CGG UGC UUU UU | -92 | Forward |
| **gRNA L** | CAU GGC CCG AGA GCU GCA UCG UUU UAG AGC UAG GCC AAC AUG AGG AUC ACC CAU GUC UGC AGG GCC UAG CAA GUU AAA AUA AGG CUA GUC CGU UAU CAA CUU GGC CAA CAU GAG GAU CAC CCA UGU CUG CAG GGC CAA GUG GCA CCG AGU CGG UGC UUU UU | -144 | Forward |
| **gRNA O** | UCU ACA AGG GAC UUU CCG CUG UUU UAG AGC UAG GCC AAC AUG AGG AUC ACC CAU GUC UGC AGG GCC UAG CAA GUU AAA AUA AGG CUA GUC CGU UAU CAA CUU GGC CAA CAU GAG GAU CAC CCA UGU CUG CAG GGC CAA GUG GCA CCG AGU CGG UGC UUU UU | -91 | Forward |
| **gRNA scr** | GCA CUA CCA GAG CUA ACU CAG UUU UAG AGC UAG GCC AAC AUG AGG AUC ACC CAU GUC UGC AGG GCC UAG CAA GUU AAA AUA AGG CUA GUC CGU UAU CAA CUU GGC CAA CAU GAG GAU CAC CCA UGU CUG CAG GGC CAA GUG GCA CCG AGU CGG UGC UUU UU | N/A | N/A |

**Table S3. Formulation parameters of patisiran LNP and LNP X**.

|  | **Patisiran LNP** | **LNP X** |
| --- | --- | --- |
| **Ionizable lipid (relative molar ratio)** | DLin-MC3-DMA (50) | SM-102 (50) |
| **Helper lipid (relative molar ratio)** | DSPC (10) | DSPC (10) |
| **Sterol (relative molar ratio)** | Cholesterol (38.5) | ß-sitosterol (38.5) |
| **PEG lipid (relative molar ratio)** | DMG-PEG2000 (1.5) | DMG-PEG2000 (1.5) |
| **Lipid concentration** | 10 mM | 10 mM |
| **Flow rate ratio (aqueous : organic)** | 1.8 : 1 | 1.8 : 1 |
| **N/P ratio** | 6 | 6 |

#
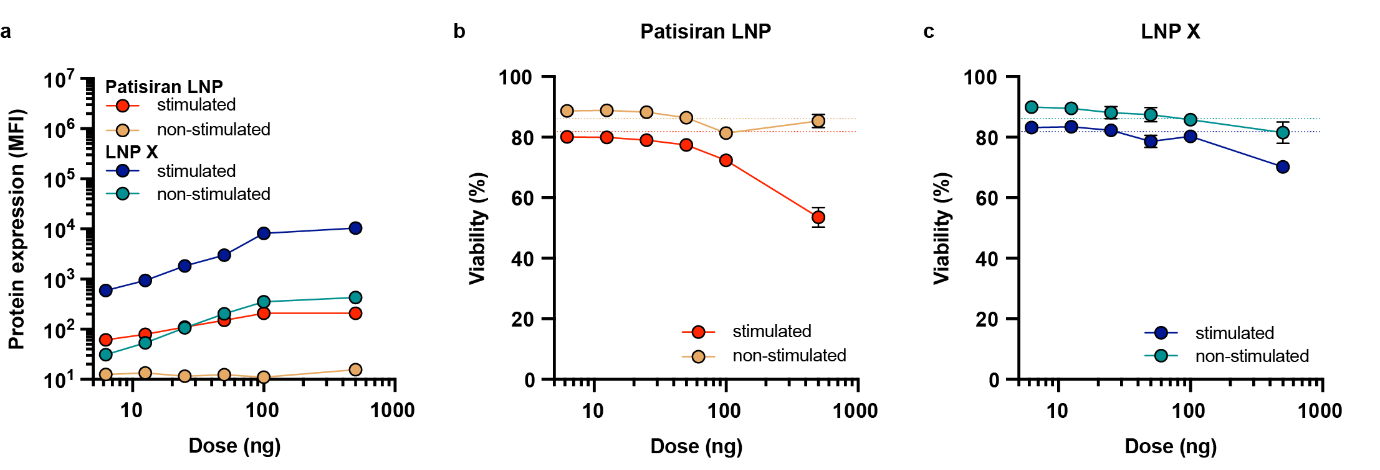
Figure S1. Treatment of CD4^+^ T cells with LNP X results in greater protein expression and lower toxicity compared to patisiran LNP. CD4^+^ T cells from HIV-negative donors were rested or pre-stimulated with anti-CD3/anti-CD28, then treated with patisiran LNP or LNP X encapsulating mCherry mRNA for 72h at the indicated doses. (a) Protein expression was quantified by the median fluorescent intensity (MFI) of mCherry within live cells. (b,c) Cellular toxicity was determined by LIVE/DEAD stain using flow cytometry. Dotted lines represent baseline viability in the absence of LNP treatment. (a-c) Mean ± SEM, n=6 donors.

**
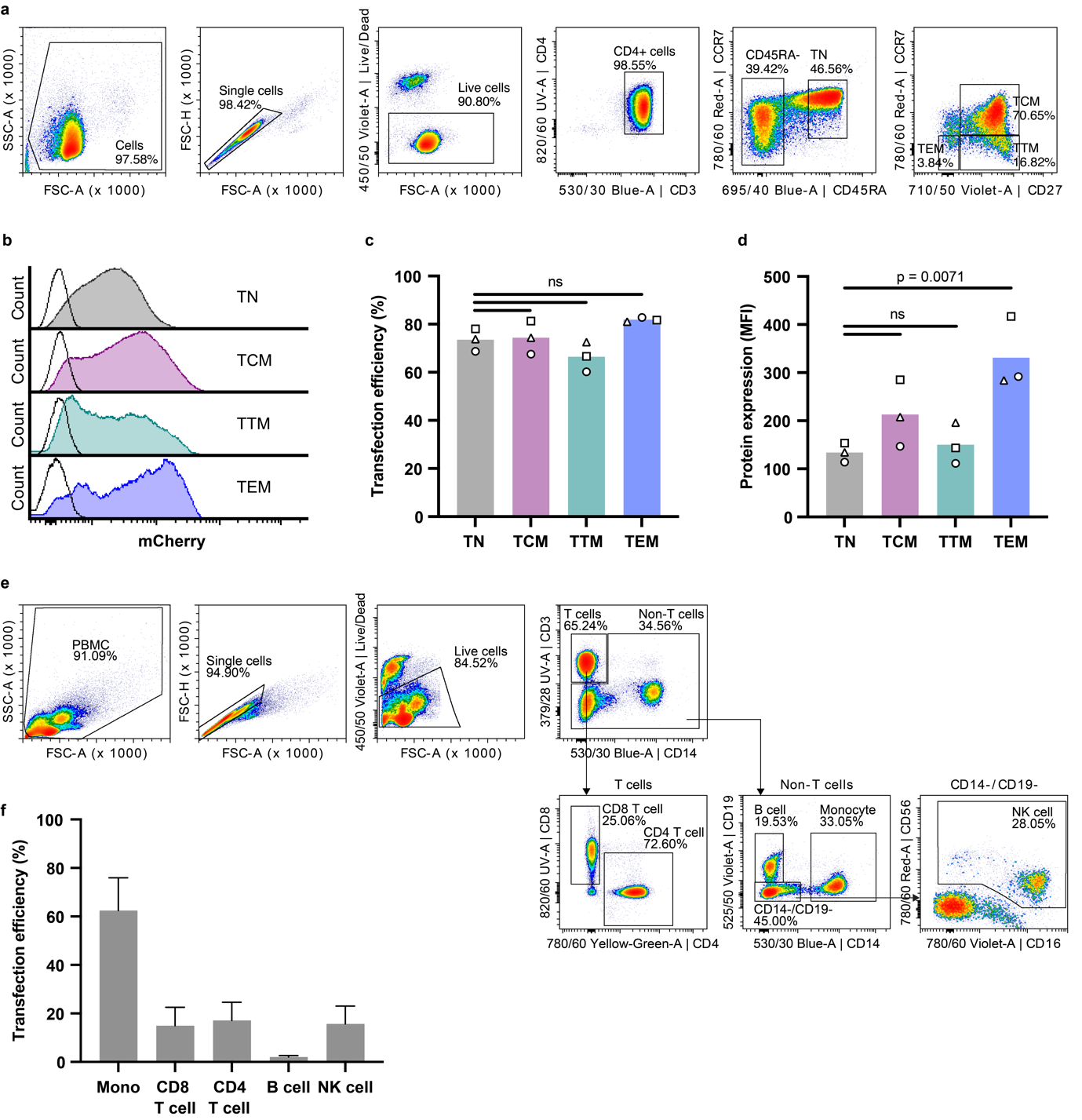
**

**Figure S2. Uptake of LNP X and expression of mRNA in CD4^+^ T cell subsets and in CD4^+^ T cells in the context of peripheral blood mononuclear cells (PBMCs). (a-d)** Non-stimulated CD4^+^ T cells from HIV-negative donors were treated with 50 ng LNP X encapsulating mCherry mRNA for 72h. Naïve (TN), central memory (TCM), transitional memory (TTM) and effector memory (TEM) subsets were defined by flow cytometry. (a) Representative gating strategy. (b) Representative histograms of mCherry expression in each subset (filled histograms) compared to background fluorescent in non-treated cells (black line). (c) Transfection efficiency of mCherry-LNP X in each T cell subset as defined by the percentage of live cells expressing mCherry. (d) Protein expression was quantified by the median fluorescent intensity (MFI) of mCherry within each T cell subset. (c,d) Bars represent means of n=3 donors. Significance was determined using a paired t test in (c) or a ratio paired t test in (d) to allow comparisons of MFI values between experiments, ns non-significant. (e,f) PBMCs from HIV-negative donors were treated with 200 ng LNP X encapsulating mScarlet mRNA for 24h. Antibodies to detect CD4+ T cells (CD3+CD4+), monocytes (Mono; CD14+), CD8+ T cells (CD3+CD8+), B cells (CD19) and natural killer (NK, CD56+CD16+) cells. (e) Representative gating strategy. (f) Percentage of live cells expressing mScarlet within each cell subset. Mean ± SEM, n=6 donors.


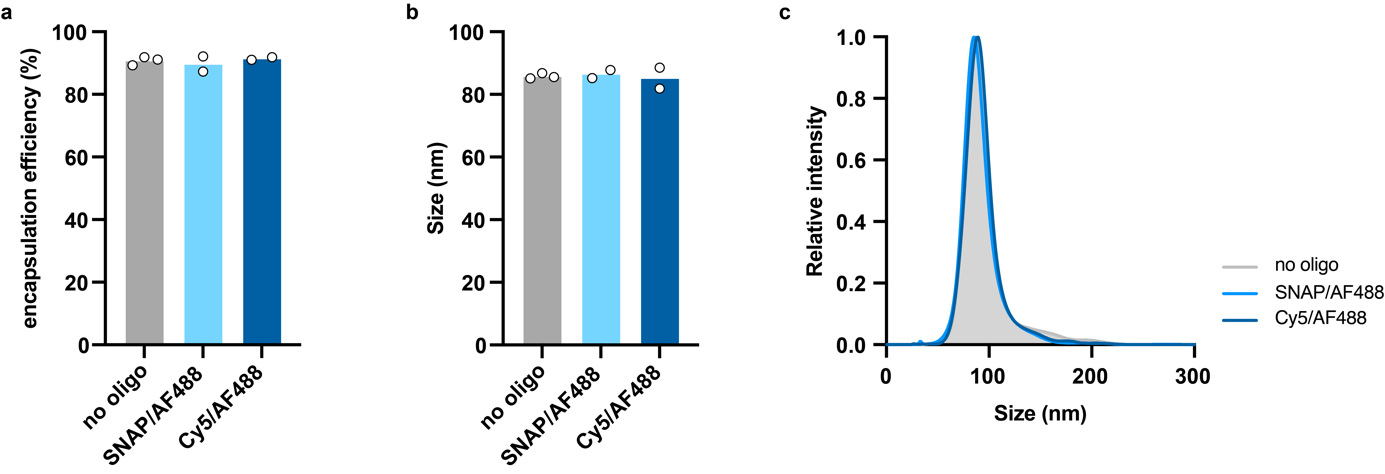


**Figure S3. SNAP_switch_ LNPs exhibit similar physicochemical characteristics to single mRNA-encapsulating LNPs.** (a-c) LNPs (n=2-3) were formulated according to the LNP X formulation, encapsulating either only an mRNA encoding mScarlet (no oligo), or an mRNA encoding mScarlet in addition to the SNAP_switch_ oligo (SNAP) or Cy5 oligo and AF488 oligo. Encapsulation efficiency of the resulting LNPs was determined by a modified RiboGreen assay (a) and the size (b) and size distribution (c) were determined using Nanoparticle Tracking Analysis.

#
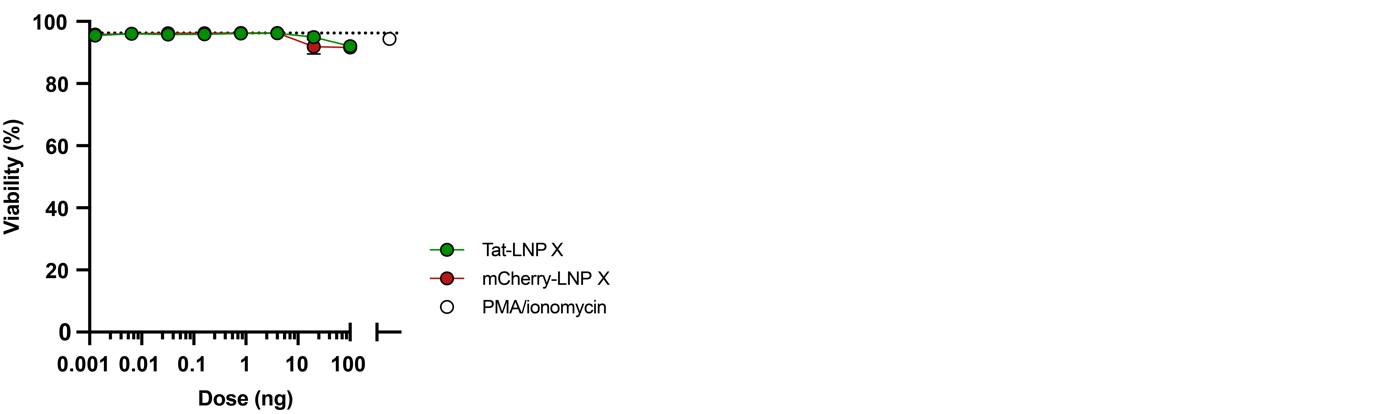


# Figure S4. Treatment of J-Lat 10.6 cells with Tat- or control LNP X is not toxic. J-Lat 10.6 cells were treated for 24h with indicated doses of LNP X encapsulating mRNA expressing the 72 amino acids of the first coding exon of HIV Tat (Tat-LNP X) or mCherry (mCherry-LNP X) as control. Cellular toxicity was determined after 24h by viability staining using flow cytometry. Mean ± SEM, n=3 independent experiments. Dotted line represents baseline viability in the absence of LNP treatment.

**
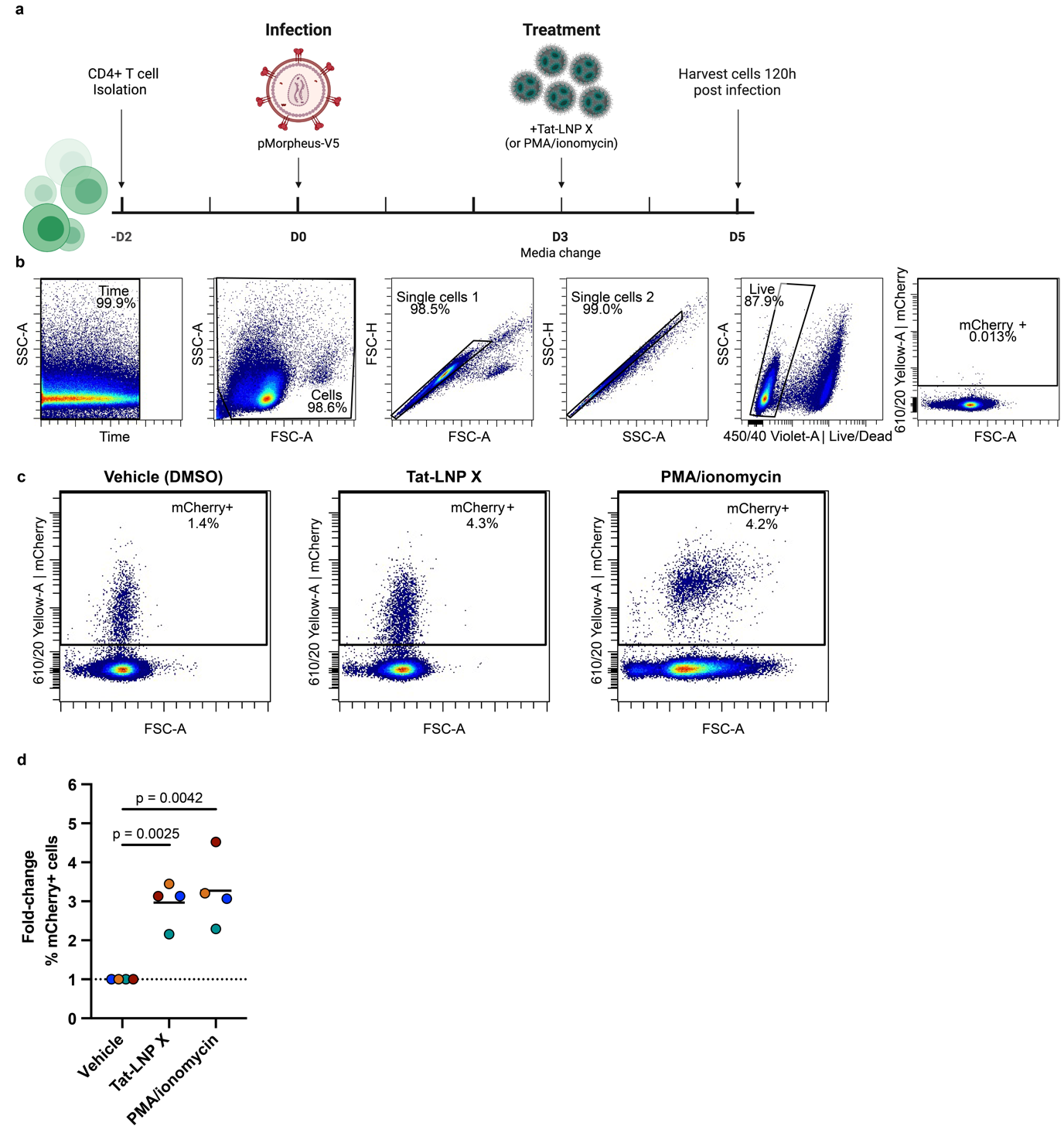
Figure S5. Tat-LNP X reactivate HIV in a primary cell model of infection.** CD4^+^ T cells from HIV-negative donors were infected with pMorpheus-V5 reporter virus. 3 days post-infection, cells were treated with Tat-LNP X, PMA/ionomycin (positive control) or DMSO (vehicle control for PMA/ionomycin). (a) Schematic of experimental setup. Created in BioRender: *Cevaal, P. (2025) https://BioRender.com/8e7ui7f.* (b) Gating strategy showing uninfected cells. Cells were gated on time, FSC/SSC, single cells and Live/Dead negative (live cells) before calculating the percentage mCherry+ cells as a measure of productive infection. (c) Representative flow plots showing the population of productively infected cells. (d) Fold-change of live cells expressing mCherry compared to vehicle control as a measure of the induction of productive infection. Lines represent mean of n=4 donors. Significance was determined using a paired t test.

#
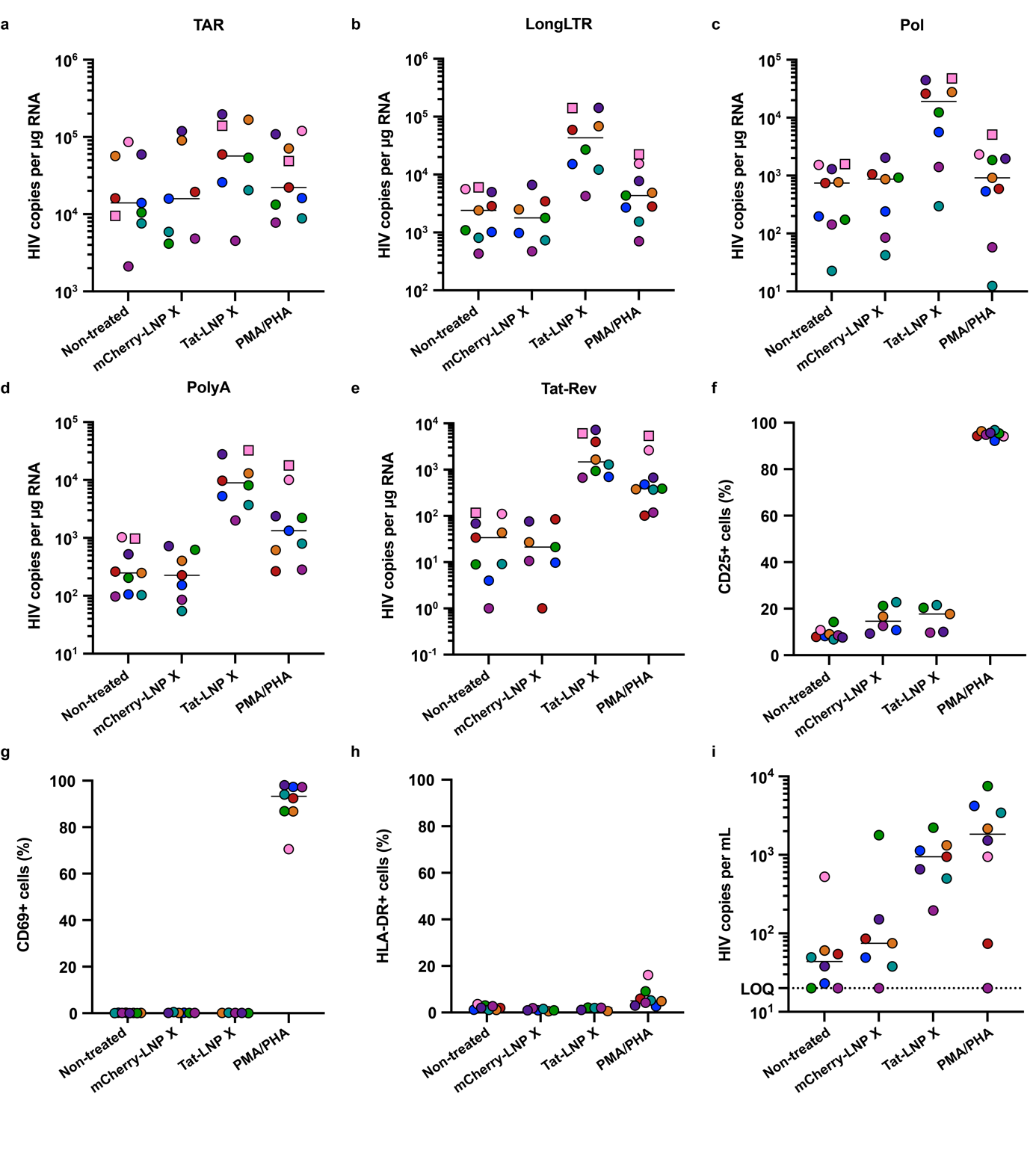


# Figure S6. Tat-LNP X induce the expression of all measured HIV transcripts *ex vivo*. CD4^+^ T cells from people living with HIV on suppressive ART were treated with 200 ng Tat-LNP X, mCherry-LNP X per 10^5^ cells (4 µg/mL) or PMA/PHA as a positive control as in Figure 2. After 48h (squares) or 72h (circles), expression of HIV transcripts TAR (a) LongLTR (b), Pol (c), PolyA (d) and Tat-Rev (e) representing transcription initiation, proximal elongation, distal elongation, completion and splicing, respectively, was determined using digital RT-PCR. HIV copies per μg RNA (to account for differences in RNA input) is shown. Concurrently, the expression of cellular activation markers CD25 (f), CD69 (g) and HLA-DR (h) was determined using flow cytometry. Where datapoints are missing in panel f-h, cell input was insufficient to perform an accurate measurement. (i) After 72h, the number of copies of HIV RNA per mL of supernatant was quantified using RT-PCR. Short horizontal line represents the median of n=7-8 donors. LOQ, limit of quantification.

#
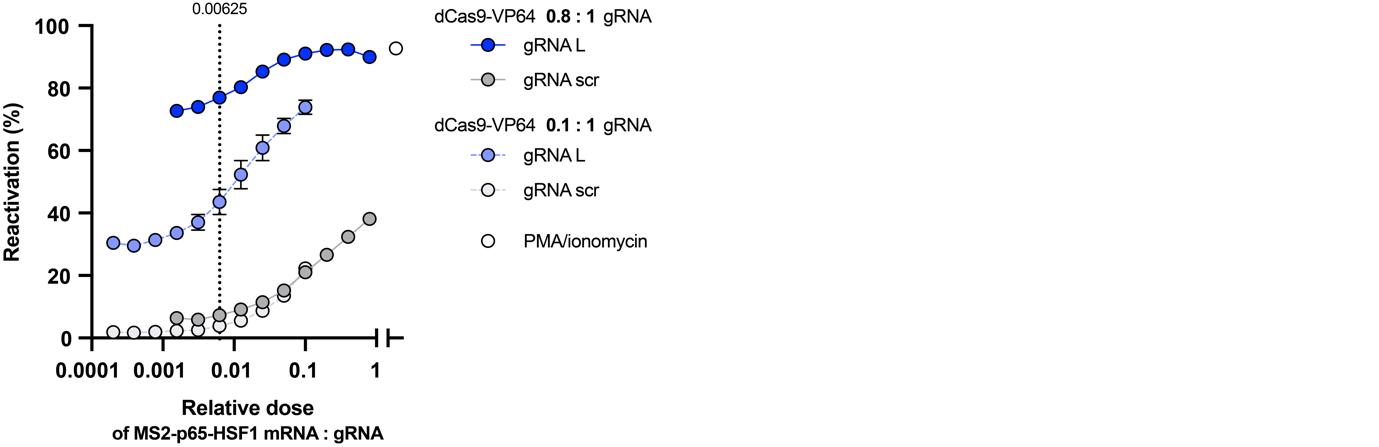


# Figure S7. Titration of CRISPR activation machinery components. J-Lat A2 is a T cell line that expresses GFP under the control of the HIV LTR promoter. J-Lat A2 cells were simultaneously treated with three LNP X formulations containing either dCas9-VP64 mRNA, MS2-p65-HSF1 mRNA or HIV LTR-targeting gRNA L (as used in subsequent HIV studies, see also Figure 5) or scrambled (scr) control gRNA, respectively. The gRNA-LNP X dose was kept constant at 64 ng per 10^5^ cells; dCas9-VP64-LNP X was dosed at either 64 ng (dark blue/grey) or 8 ng (light blue/grey), representing a mass ratio of 0.8 : 1 or 0.1 : 1 dCas9-VP64 : gRNA, respectively. The MS2-p65-HSF1-LNP X dose was titrated to identify the ratio of the CRISPRa machinery components that yielded the highest potency (gRNA L, blue) with lowest non-specific activation (gRNA scr, grey). After 24h, CRISPRa potency was determined by measuring GFP using flow cytometry, as a marker of reactivation of HIV LTR-mediated transcription. PMA/ionomycin treatment was included as a positive control. Mean ± SEM, n=2 independent experiments.

#
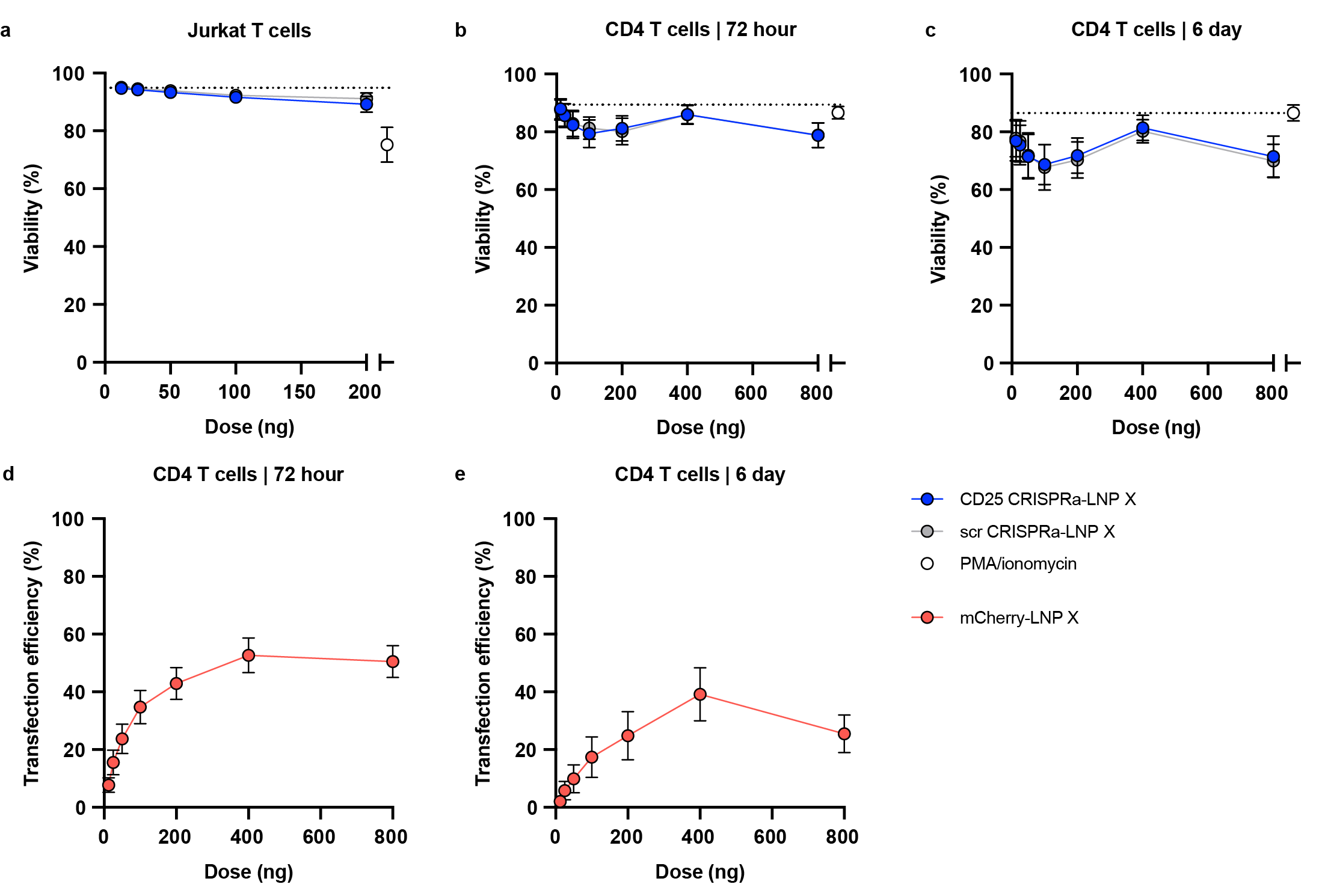
Figure S8. CRISPRa-LNP X-mediated induction of endogenous gene expression is non-toxic in T cell lines and primary T cells. (a-c) LNP formulation X was used to encapsulate the CRISPR activation machinery (CRISPRa-LNP X) including a gRNA targeting the endogenous gene encoding CD25 (CD25 CRISPRa-LNP X) or a scrambled control gRNA (scr CRISPRa-LNP X) as in Figure 4. LNPs were added to Jurkat T cells for 24h (a) or primary CD4^+^ T cells for 72h (b) or 6 days (c), after which viability was determined using flow cytometry. An mCherry-LNP X control was included to assess the transfection efficiency at 72h and 6 days of culture (d,e). Means ± SEM for n=4 independent experiments (a) or n=5-6 donors (b-e). Dotted lines represent baseline viability in the absence of LNP treatment.

#
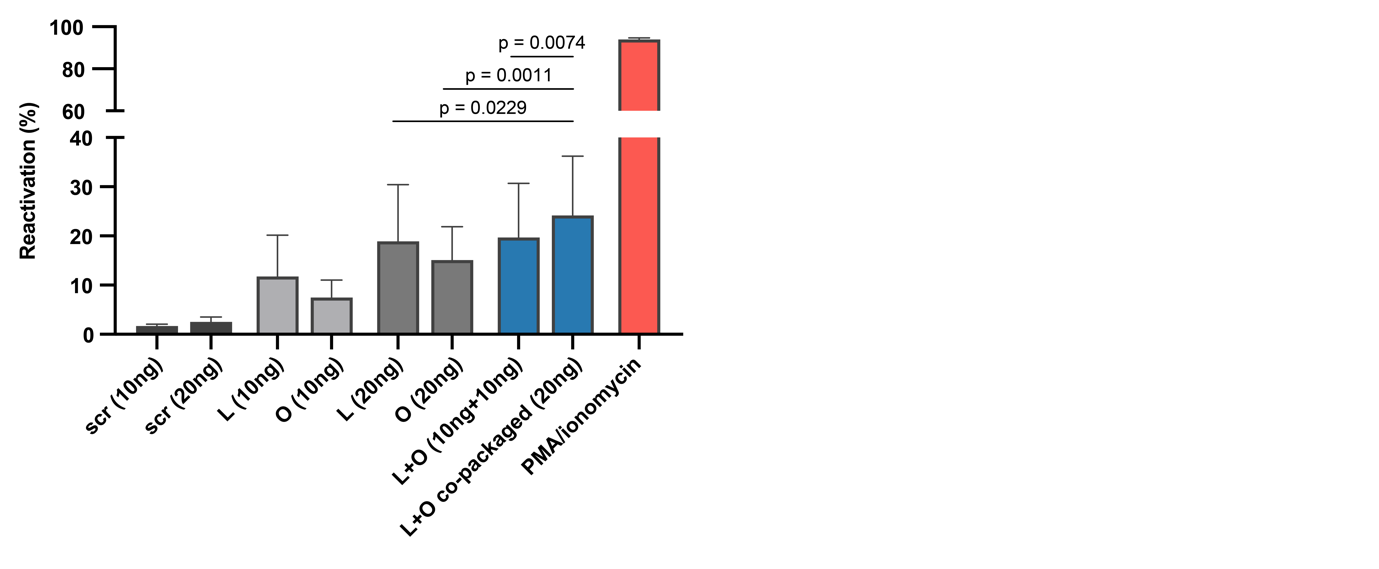
Figure S9. Co-packaging of two HIV LTR-targeting gRNAs enhances CRISPRa potency. J-Lat A2 cells were treated with sub-optimal doses of CRISPRa-LNP X containing gRNA L, O, or scrambled (scr), a 1:1 mixture of two CRISPRa-LNP X formulations containing gRNA L and O, respectively (L+O), or CRISPRa-LNP X containing a 1:1 mixture of gRNAs L and O (L+O co-packaged). Potency to reactivate HIV LTR-mediated transcription was determined using flow cytometry and compared using a ratio paired t test. Mean ± SEM, n=5 independent experiments.

#
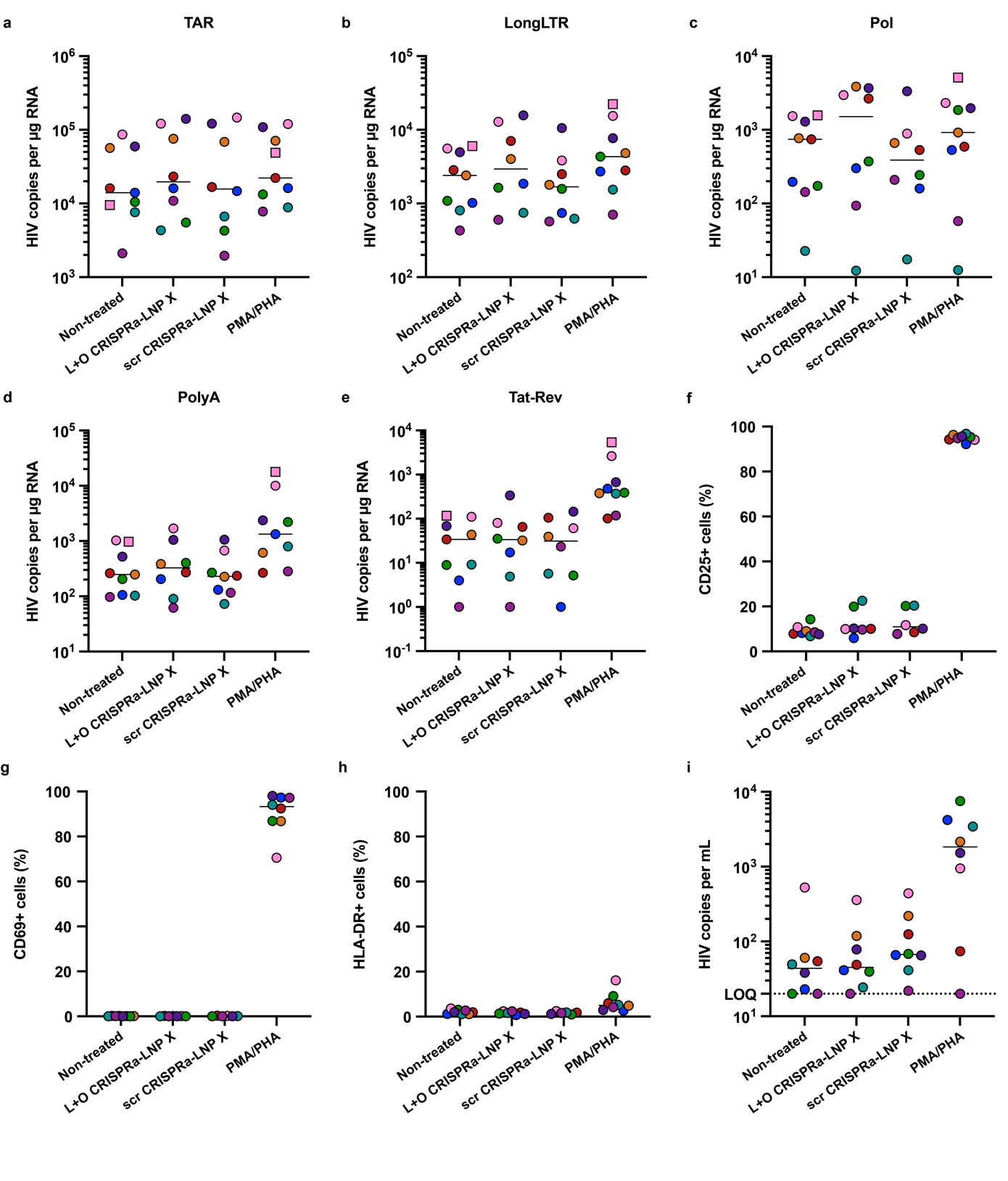


# Figure S10. CRISPRa-LNP X induce the expression of early HIV transcripts *ex vivo*. CD4^+^ T cells from people living with HIV on suppressive ART were treated with 200 ng CRISPRa-LNP X harbouring gRNAs L and O (L+O CRISPR-LNP X) or a scrambled gRNA (scr CRISPRa-LNP X) per 10^5^ cells (4 µg/mL) or PMA/PHA as a positive control. After 48h (squares) or 72h (circles), expression of HIV transcripts TAR (a) LongLTR (b), Pol (c), PolyA (d) and Tat-Rev (e) representing transcription initiation, proximal elongation, distal elongation, completion and splicing, respectively, was determined using digital RT-PCR. HIV copies per μg RNA (to account for differences in RNA input) is shown. Concurrently, the expression of cellular activation markers CD25 (f), CD69 (g) and HLA-DR (h) was determined using flow cytometry. Where datapoints are missing in panel f-h, cell input was insufficient to perform an accurate measurement. (i) After 72h, the number of copies of HIV RNA per mL of supernatant was quantified using RT-PCR. Short horizontal line represents the median of n=7-8 donors. LOQ, limit of quantification.
