# Supplementary material for: Efficient mRNA delivery to resting T cells to reverse HIV latency
Source: Nat Commun. 2025 May 29;16:4979. doi: 10.1038/s41467-025-60001-2 (PMC12122926; doi:10.1038/s41467-025-60001-2)
Supplement: Supplementary file 3 — Reporting Summary [file 41467_2025_60001_MOESM3_ESM.pdf]

nature portfolio

Corresponding author(s): Sharon R Lewin

Last updated by author(s): Mar 31, 2025

Reporting Summary

Nature Portfolio wishes to improve the reproducibility of the work that we publish. This form provides structure for consistency and transparency in reporting. For further information on Nature Portfolio policies, see our Editorial Policies and the Editorial Policy Checklist.

Statistics

For all statistical analyses, confirm that the following items are present in the figure legend, table legend, main text, or Methods section.

n/a	Confirmed	
☐	☒	The exact sample size (*n*) for each experimental group/condition, given as a discrete number and unit of measurement
☐	☒	A statement on whether measurements were taken from distinct samples or whether the same sample was measured repeatedly
☐	☒	The statistical test(s) used AND whether they are one- or two-sided<br>*Only common tests should be described solely by name; describe more complex techniques in the Methods section.*
☐	☒	A description of all covariates tested
☐	☒	A description of any assumptions or corrections, such as tests of normality and adjustment for multiple comparisons
☐	☒	A full description of the statistical parameters including central tendency (e.g. means) or other basic estimates (e.g. regression coefficient) AND variation (e.g. standard deviation) or associated estimates of uncertainty (e.g. confidence intervals)
☐	☒	For null hypothesis testing, the test statistic (e.g. *F*, *t*, *r*) with confidence intervals, effect sizes, degrees of freedom and *P* value noted<br>*Give P values as exact values whenever suitable.*
☒	☐	For Bayesian analysis, information on the choice of priors and Markov chain Monte Carlo settings
☒	☐	For hierarchical and complex designs, identification of the appropriate level for tests and full reporting of outcomes
☒	☐	Estimates of effect sizes (e.g. Cohen's *d*, Pearson's *r*), indicating how they were calculated

Our web collection on statistics for biologists contains articles on many of the points above.

Software and code

Policy information about availability of computer code

Data collection	Flow cytometry data was collected using BD FACSDivaTM software (version 9.0) or SpectroFlo (version 3.3.0).<br>LNP size and polydispersity indices were collected using ZS Xplorer software (Malvern Panalytical, version 3.3.0.42).<br>RiboGreen fluorescence values were collected using Omega and MARS Data Analysis Software (BMG LABTECH, version 3.32 R5).<br>Digital PCR data were collected using QIAcuity Software suite (Qiagen, version 2.5.0.1)
Data analysis	Flow cytometry data were analysed using the Omiq software from Dotmatics (www.omiq.ai, www.dotmatics.com), Flowjo 10.9, FCS Express 7 (De Novo Software) and R 4.2.3.<br>Graphics were made and statistic calculations were performed using GraphPad Prism 10.2.3

For manuscripts utilizing custom algorithms or software that are central to the research but not yet described in published literature, software must be made available to editors and reviewers. We strongly encourage code deposition in a community repository (e.g. GitHub). See the Nature Portfolio guidelines for submitting code & software for further information.

Data

Policy information about availability of data

All manuscripts must include a data availability statement. This statement should provide the following information, where applicable:

- Accession codes, unique identifiers, or web links for publicly available datasets
- A description of any restrictions on data availability
- For clinical datasets or third party data, please ensure that the statement adheres to our policy

All data relevant for the conclusions in this manuscript are available in the article or extended data. Source data are provided with the manuscript. Any additional data are available from the corresponding author upon reasonable request.

Research involving human participants, their data, or biological material

Policy information about studies with human participants or human data. See also policy information about sex, gender (identity/presentation), and sexual orientation and race, ethnicity and racism.

Reporting on sex and gender	This study includes a total of n=8 people living with HIV on stable antiretroviral therapy. All of these people are male which is a direct reflection of the population of people living with HIV in Australia, the majority of which consists of men who have sex with men.
Reporting on race, ethnicity, or other socially relevant groupings	Race/ethnicity/social groups are not reported in this study.
Population characteristics	Median age: 56 years (IQR: 52.75-66.25)<br>Median ART duration: 10.65 years (IQR: 6.25-11.48)
Recruitment	People living with HIV on antiretroviral therapy were recruited with the following inclusion criteria:<br>Age >18 years of age<br>Plasma HIV RNA < 50 copies/mL for at least 2 years
Ethics oversight	Ethics were approved by Human Research Ethics committees at the Alfred Hospital in Melbourne, the University of Melbourne, and the Institutional Review Board at UCSF.

Note that full information on the approval of the study protocol must also be provided in the manuscript.

Field-specific reporting

Please select the one below that is the best fit for your research. If you are not sure, read the appropriate sections before making your selection.

[x] Life sciences   [ ] Behavioural & social sciences   [ ] Ecological, evolutionary & environmental sciences

For a reference copy of the document with all sections, see nature.com/documents/nr-reporting-summary-flat.pdf

Life sciences study design

All studies must disclose on these points even when the disclosure is negative.

Sample size	Sample sizes for experiments using cell lines or primary cells from HIV-negative donors were based on standard practice in the field. Sample sizes for experiments using cells from people living with HIV (ex vivo) were calculated a priori based on an expected >3.5-fold increase in HIV RNA which would require n=7 evaluable participants to provide 80% statistical power at alpha=0.05. As 10-20% of participants may not respond to the stimulus based on previous work (PMID 26614966, 25393648, 26423811), n=8 participants were evaluated in this work.
Data exclusions	No data were excluded from analysis.
Replication	Cell line experiments were performed using two technical replicates within each of the n=3 independent experiments. Technical replicates showed minimal differences and were averaged to generate one dataset per independent experiment. Experiments with primary cells from HIV-negative donors showed good replicability between donors and no datasets were excluded due to lack of replicability. Digital PCR analysis of HIV RNA transcripts from ex vivo cells from people living with HIV was performed in four technical replicate wells per sample which showed good replicability. Replicate wells were averaged and no replicates were excluded due to lack of replicability.
Randomization	Not applicable
Blinding	Not applicable

Reporting for specific materials, systems and methods

We require information from authors about some types of materials, experimental systems and methods used in many studies. Here, indicate whether each material, system or method listed is relevant to your study. If you are not sure if a list item applies to your research, read the appropriate section before selecting a response.

Materials & experimental systems

n/a	Involved in the study
	[x] Antibodies
	[x] Eukaryotic cell lines
[x]	Palaeontology and archaeology
[x]	Animals and other organisms
[x]	Clinical data
[x]	Dual use research of concern
[x]	Plants

Methods

n/a	Involved in the study
[x]	ChIP-seq
	[x] Flow cytometry
[x]	MRI-based neuroimaging

Antibodies

Antibodies used

All antibodies used are described in the Methods section of the manuscript, and summarized below:

For phenotyping using flow cytometry:
PE/Cyanine7 anti-human CD25 (Clone BC96; BioLegend). Dilution: 1/100
Brilliant Ultra VioletTM 395 anti-human CD69 (Clone FN50, Invitrogen). Dilution: 1/100
FITC anti-human HLA-DR (Clone L243, Invitrogen). Dilution: 5/23
FITC anti-human CD3 (Clone UCHT1, BD Biosciences). Dilution: 1/10
BUV805 anti-human CD4 (Clone SK3, BD Biosciences). Dilution: 1/10
PerCP/Cy5.5 anti-human CD45RA (Clone HI100, BioLegend). Dilution: 1/10
APC/Cy7 anti-human CCR7 (Clone C043H7, BioLegend). Dilution: 1/100
BV711 anti-human CD27 (Clone L128, BD Biosciences). Dilution: 1/100
FITC anti-human CD14 (Clone M5E2, BD Biosciences). Dilution: 1/30
APC/Cy7 anti-human CD56 (Clone HCD56, BioLegend). Dilution: 1/30
BUV395 anti-human CD3 (Clone UCHT1, BD Biosciences). Dilution: 0.7/30
BUV805 anti-human CD8 (Clone SK1, BD Biosciences). Dilution: 0.3/30
BV510 anti-human CD19 (Clone HIB19, BioLegend). Dilution: 1/30
BV650 anti-human HLA-DR (Clone G46-6, BD Biosciences). Dilution: 1/30
BV785 anti-human CD16 (Clone 3G8, BioLegend). Dilution: 1/30
PE/Cy7 anti-human CD4 (Clone OKT4, BioLegend). Dilution: 0.3/30

For T cell activation in vitro:
Ultra-LEAF™ Purified anti-human CD3 Antibody (Clone OKT3, Biolegend)
Ultra-LEAF™ Purified anti-human CD28 Antibody (Clone CD28.2, Biolegend)

Validation

All antibodies were validated by the respective manufacturers to be reactive to human antigen and were validated for the method of use employed in this work. Fluorescent antibodies used for phenotyping were further validated in-house through titrations.

Eukaryotic cell lines

Policy information about cell lines and Sex and Gender in Research

Cell line source(s)	J-Lat A2 (RRID:CVCL_1G43) and J-Lat 10.6 (RRID:CVCL_8281) are derived from Jurkat T cells, which originates from a male donor with T cell leukemia. Cell lines were obtained through the NIH AIDS Reagent Program.
Authentication	Cell lines were authenticated by the NIH AIDS Reagent Program before distribution. No further authentication was performed in-house.
Mycoplasma contamination	Cell lines were not tested for mycoplasma contamination.
Commonly misidentified lines (See ICLAC register)	No commonly misidentified cell lines were used in this work.

Plants

Seed stocks	NA
Novel plant genotypes	NA
Authentication	NA

Flow Cytometry

Plots

Confirm that:

☒ The axis labels state the marker and fluorochrome used (e.g. CD4-FITC).

☒ The axis scales are clearly visible. Include numbers along axes only for bottom left plot of group (a 'group' is an analysis of identical markers).

☒ All plots are contour plots with outliers or pseudocolor plots.

☒ A numerical value for number of cells or percentage (with statistics) is provided.

Methodology

Sample preparation	After treatment with LNPs, cells were collected by centrifugation for 4 minutes at 400 g, then washed three times in PBS prior to staining with LIVE/DEAD Fixable Violet Dead Cell Stain Kit as per the Methods section. Cells were then washed three times in PBS with 1% fetal bovine serum and 1 µM EDTA before further phenotyping (see Methods) or fixation using PBS with 1% paraformaldehyde.
Instrument	BD LSR II Fortessa flow cytometer (BD Biosciences) or Cytek Aurora Spectral cytometer (Cytek)
Software	Flow cytometry data were analysed using the Omiq software from Dotmatics (www.omiq.ai, www.dotmatics.com), Flowjo 10.9 and FCS Express 7 (De Novo Software).
Cell population abundance	No cell populations were sorted during this work.
Gating strategy	Unless specified otherwise and exemplified using a gating strategy, flow cytometry data was gated as follows:<br>Based on a FSC-A/SSC-A plot, cells were gated from debris. Using a FSC-A/FSC-H plot, single cells were gated from doublet. Viable cells were subsequently identified as the population of cells staining negative for Live/Dead viability dye (see Methods). Subsequent gates were set using FMO controls.

☒ Tick this box to confirm that a figure exemplifying the gating strategy is provided in the Supplementary Information.
